# Contraceptive technologies for global health: ethically getting to safe, effective and acceptable options for women and men

**DOI:** 10.1007/s13346-020-00726-3

**Published:** 2020-03-02

**Authors:** John Townsend, Régine Sitruk-Ware, Saumya RamaRao, Jim Sailer

**Affiliations:** Population Council, New York, NY USA

**Keywords:** Client needs, Contraceptives, Product development and introduction, Respect, Beneficence, Reproductive justice

## Abstract

While the contributions of science, biomedicine, and engineering to contraceptive development offer wonder and promise to the community, what inspires many of us in the not-for-profit sector about the process of contraceptive product development is the integration of consultations with users, providers and policy makers, good clinical and manufacturing practice in product design and development, and the delivery of approved products at affordable prices to those in greatest need. The commitment to have an impact on the reproductive lives of women and men along with the ethical principles embedded in this process of achieving safe, effective, and acceptable options include the respect for persons, i.e., eventual users, beneficence for those using the product and justice in ensuring that it is available to those who are most vulnerable, including those in developing countries. It is the inspiration that drives the scientists and developers to produce public benefit and additional social value.

Worldwide every year, there are about approximately 85 million unintended pregnancies. An important reason is that existing contraceptive technologies (see Fig. 1) do not always meet the needs or preferences of women and men. Many are not effective enough (< 98%), they may have undesirable side effects, or the requirements for use do not fit into the lives of users and their families.

**Fig. 1:**
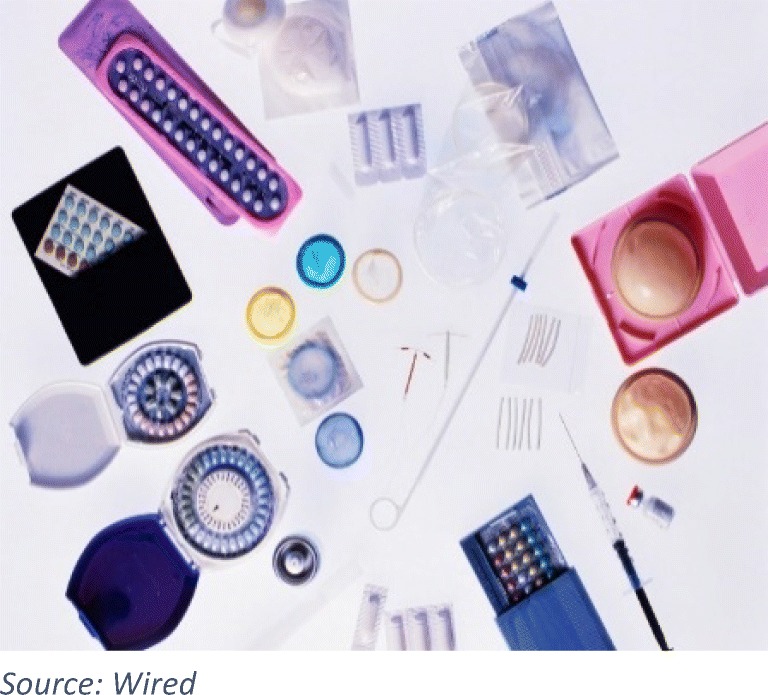
Common contraceptive options. Source: wired

The Population Council as an international NGO has as its mission to develop contraceptive technologies that contribute to the wellbeing of humanity globally. Through its Center for Biomedical Research, the Council has developed and licensed some of the most widely used long-acting, reversible contraceptives in the world. Currently, 170 million women worldwide are using a highly effective contraceptive developed by the Population Council or based on our technology, including the Copper T intrauterine device; Mirena®, the levonorgestrel-releasing intrauterine system; the implants Jadelle® and Norplant®, the Progering™ contraceptive vaginal ring, and most recently a contraceptive vaginal system, Annovera™.

In the past 30 years, two demands have been raised by potential clients about female contraception: the need for long-acting reversible options as an alternative to permanent methods such as tubectomy, and the desire for short- to mid-term discrete products under the client’s control that provide the level of protection comparable to pills and injectables. The two products recently developed by the Council that meet these requirements were the Mirena™ hormonal 5-year intrauterine system (LNG IUS) and the Annovera™ 1-year contraceptive vaginal system. The IUS is a long-acting reversible method which is dependent on a skilled health provider for insertion and removal, and the vaginal system is a mid-acting reversible easy-to-use method under the control of the user.

The first levonorgestrel intrauterine system with a high level of effectiveness (> 98%) and the benefit of protection against heavy menstrual bleeding (LNG IUS with the brand name Mirena™, see Fig. 2) was developed by the Population Council and Leiras Pharma in Turku, Finland [1]. It was first registered in Finland in 1990, is produced by Bayer AG in Turku, was approved by the US Food and Drug Administration (US FDA) in 2000, and marketed by Bayer AG worldwide. The non-branded LNG IUS has been donated to public sector organizations in developing countries by the Finnish registered ICA Foundation since 2004.

**Fig. 2:**
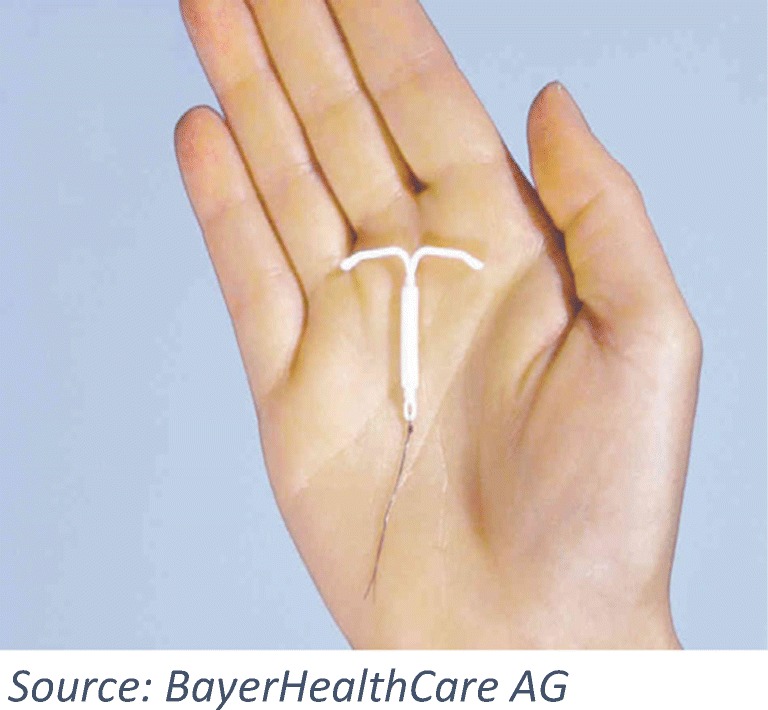
Mirena™ intrauterine system (LNG IUS). Source: BayerHealthCare AG

ANNOVERA™ (see Fig. 3) is a ring-shaped, nonbiodegradable, flexible, opaque white vaginal system containing two active ingredients, a novel progestin (Nestorone®) and an estrogen, which is highly effective in the prevention of pregnancy (> 97%) [2, 3]. Annovera™ was approved by the US FDA in 2018, is produced in Malmö, Sweden by QPharma, and distributed by TherapeuticsMD in the USA.

**Fig. 3:**
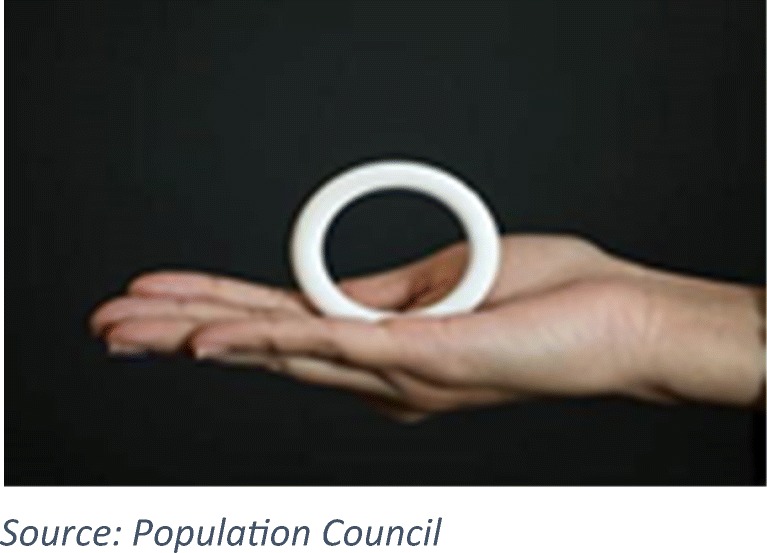
Annovera™ vaginal system. Source: Population Council

While it may seem easy in hindsight, getting to this point is a long, risky process that mobilizes state-of-the-art scientific knowledge while capturing the needs and preferences of multiple groups: potential clients, product developers and health system professionals, policy makers and regulators, and public sector sponsors, i.e., NICHD, USAID, and WHO. We can conceptualize the process as an interactive one between the principles of medical ethics and the classic phases of pharmaceutical research, i.e., from pre-clinical to regulatory approval, through clinical phases I–III, and phase IV post-marketing surveillance. The key is to ensure that the commitments to the principles of medical ethics [4] are not overlooked, but rather are an integral part of the development process. To be successful, the interface must be reviewed periodically from the development of a target product profile to the introduction and surveillance of the product in actual markets.

The first commitment is an expression of the first principle of respect. While this principle is often linked to informed consent, it really is about respect for the user, their autonomy to make decisions about their health, and their preferences. It is the commitment to listen, understand and learn from women or men who want to use contraception but are currently not satisfied with any product on the market or are not using any method at all. This can be up to 40% in developing countries and about 20% in more developed markets. We first have to seek the advice, guidance, and preferences of multiple samples of intended users. If a diverse set of clients are not consulted, informed of possible features of a new product and the strength of their preferences and trade-offs not taken into account, it is unlikely that the end product will be in high demand in target markets. These products were developed with the intention of serving both young and older users, those with easy access to health systems as well as those who would only need to seek care at first adoption or when they want to switch to another method. User-centered design thinking must be incorporated at every stage of product development and introduction.

The second commitment involves the principle of beneficence or the pursuit of quality or production of good rather than risk or harm to the user. Here, the focus is on ensuring that the product as designed and amended over time meets the needs of potential clients in a safe, effective, acceptable, and increasingly affordable fashion. This commitment ensures that the perspectives of users are incorporated into prototype designs with available technology, including active pharmaceutical ingredients (API), delivery systems, potential stability, and potential for safety with a minimum of predictable mild side effects. Often, early prototypes are unwieldly and frankly unviable, but the creativity of the product developers when blended with the preferences of users through user-centered design processes makes the magic happen. Part of this translational process is the requirement for moving beyond our individual expertise to create something with additional value for clients, providers, and health systems. This is also the step when the process of manufacturing can be modeled, and first estimates of the cost of the goods (COGs) prepared. This is also when we can explore what priority market segments appear ready for product introduction.

The third ethical principle is distributive justice, or the active pursuit of reproductive justice. Does the product actually benefit those in need in the markets where inequity and lack of access often prevail? This is more of a challenge that it seems, given the complex nature of health systems, regulatory process, the requirements of demand creation for new products, and market characteristics. The move to markets is a major translational step as the product moves away from the controlled environment of the laboratory and clinical trials to the real-world dynamics of clients, payers, and providers. Not everyone will welcome a new product, given the nature of competition as well as the traditions and skills of providers, and the habits of consumers. Significant effort is required to introduce a new product into each market, with diverse requirements for registration, the demands of marketing partners, the training and oversight of providers in both public and private institutions, and product support to users. This pursuit is further complicated by often complex procurement processes, supply chain conditions, and reporting obligations, e.g., post-marketing surveillance. This stage is not for the faint of heart but rather for those with a commitment to clients and providers both in the first and last mile.

It is remarkable that the process works at all given the multiple groups engaged and the time required to reach clients with a product they want to use. For the LNG IUS, the development time was about 20 years from the first pre-clinical safety studies to first regulatory approval and given the diffusion of innovations another 30 years to reach clients in Zambia or Bangladesh. For Annovera™, the comparable development time was about 25 years, and the 1-year vaginal system just began sales in the USA in 2019. Concurrently, planning efforts are underway to introduce the vaginal system in selected countries in sub-Saharan Africa. For most innovative products, issues of procurement volume and price, distribution networks, and market demand slow the effective entry to new markets. These time frames may be shortened with strategic partnerships, better sharing of data on APIs, smarter development financing for R&D, and earlier engagement with health systems and providers in target countries. Nonetheless, it is the pursuit of social value and responsiveness to the voices of potential users, both women and men, rather than profit that drives the Population Council’s investments.

## Conclusion

What do we know about the value produced? As a result of our efforts overtime to develop a range of approved contraceptive products, the Population Council estimates that 20% of all global contraceptive users are benefiting from products that were developed or directly influenced by the Council. New research is underway on testing a contraceptive transdermal gel for men, acknowledging the often ignored role of gender in the responsibility for pregnancy prevention [5]. What differentiates the Council from large pharmaceutical companies that also develop contraceptives is the continuous focus on innovations for the end user in developing country markets without the burden of protecting commercial portfolios or the demand for short-term returns on investment. The regulatory requirements are the same for all developers regardless of motivation, market experience, or size.

Since its introduction in the USA, the Mirena LNG IUS has reinvigorated the US market for IUDs, increasing use of devices from 2% in 2004 to nearly 12% of all contraceptive users in 2016. It responds to the needs of women for long-acting products, while depending on health providers for counseling, insertion, follow-up and removal [6]. The new Annovera™ vaginal system offers incredible benefits for women who want to have control over the method they use and will be able to access the product in pharmacies without the need for assistance for insertion or removal, and still have contraceptive protection for a full year. Given the health benefits of safe and effective birth spacing, the Copenhagen Consensus [7] estimates that for each US$1 invested in effective contraceptive produces up to US$140 in health and social benefits to the user, her family, and the country.

There is ample room for many actors in this process from public health activists in developing countries and scientists exploring new mechanisms of action to innovative manufacturers, dedicated providers, and communicators with creative market insights. What is inspiring for many is that the alignment of commitments with ethical principles eventually leads to more pregnancies that are wanted, and reproductive intentions that are fulfilled. Moreover, the associated economic and social benefits of contraceptive use allow women and men to invest in their families as well as pursue opportunities in the workforce or contribute to community leadership. For the young scientist, engaging in such product development efforts offers both the intellectual rewards of scholarship and the immense personal satisfaction of service to humanity and ultimately global health.
